# Genome-wide identification and expression analysis of the MADS-box gene family during female and male flower development in *Juglans mandshurica*

**DOI:** 10.3389/fpls.2022.1020706

**Published:** 2022-11-01

**Authors:** Hanxi Li, Yuxi Li, Xinxin Zhang, Kewei Cai, Yan Li, Qingcheng Wang, Guanzheng Qu, Rui Han, Xiyang Zhao

**Affiliations:** ^1^ State Key Laboratory of tree Genetics and Breeding, Northeast Forestry University, Harbin, China; ^2^ Jilin Provincial Key Laboratory of Tree and Grass Genetics and Breeding, College of Forestry and Grassland Science, Jilin Agricultural University, Changchun, China

**Keywords:** *Juglans mandshurica*, MADS-box gene family, genome-wide identification, female and male flowers, gene expression patterns

## Abstract

The MADS-box gene family plays a crucial role in multiple developmental processes of plants, especially in floral organ specification and the regulation of fruit development and ripening. *Juglans mandshurica* is a precious fruit material whose quality and yield are determined by floral organ development. The molecular mechanism of *J. mandshurica* female and male flower development depending on MADS-box genes remains unclear. In our study, 67 *JmMADS* genes were identified and unevenly distributed on 15 of 16 *J. mandshurica* chromosomes. These genes were divided into two types [type I (Mα, Mγ, Mδ) and type II (MIKC)]. The gene structure and motif analyses showed that most genes belonging to the same type had similar gene structures and conserved motifs. The analysis of syntenic relationships showed that MADS-box genes in *J. mandshurica*, *J. sigillata*, and *J. regia* exhibited the highest homology and great collinearity. Analysis of cis-acting elements showed that *JmMADS* gene promoter regions contained light, stress and hormone response cis-acting elements. The gene expression patterns demonstrated that 30 and 26 *JmMADS* genes were specifically expressed in the female and male flowers, respectively. In addition, 12 selected genes common to *J. mandshurica* female and male flowers were significantly upregulated at the mature stage and were used to validate the reliability of the transcriptome data using quantitative real-time PCR. This comprehensive and systematic analysis of *J. mandshurica* MADS-box genes lays a foundation for future studies on MADS-box gene family functions.

## Introduction

Transcription factors (TFs) are proteins that can bind specific cis-acting elements in promoter regions to determine gene expression levels, thereby controlling plant growth and development (Riechmann et al., 2000; Wray et al., 2003). MADS-box family genes as transcription factors (TFs), contain a highly conserved MADS domain consisting of amino acids 56–60 in the N-terminus (Schwarz-Sommer et al., 1990). The MADS domain name is based on the initials of the four transcription factors that were first discovered within this family: MINICHROMOSOME MAINTENANCE 1 (MCM1) of yeast (Sommer et al., 1990), AGAMOUS (AG) of *Arabidopsis thaliana* (Yanofsky et al., 1990), DEFICIENS (DEF) of *Antirrhinum majus* (Norman et al., 1988), and SERUM RESPONSE FACTOR (SRF) of human (Honma and Goto, 2001). Proteins composing the MADS domain are considered MADS-box proteins (Passmore et al., 1988). MADS-box family genes are widely reported in eukaryotes, including plants, animals and fungi, as preforming various biological functions, especially related to floral organ specification, flowering time regulation, fruit development and ripening (Alvarez-Buylla et al., 2000; Saedler et al., 2001).

Based on structural characteristics, the MADS-box family has been classified into two major categories: type I and type II (De Bodt et al., 2003). Type I MADS-box transcription factors in plants are SRF-like. Type II MADS-box transcription factors include the Myocyte Enhancer Factor 2-like (MEF2-like) group in animals and yeast, known as the MIKC genes in plants (Kaufmann et al., 2005). At present, the detailed study of MADS-box proteins is limited to MIKC genes, which exhibit a typical modular structure. On the basis of phylogenetic analysis and structural features, MIKC genes can be further divided into MIKC^C^ and MIKC* types, and MIKC^C^ genes have been widely reported due to the associations involved in plant growth and development (Smaczniak et al., 2012; Daminato et al., 2014).

In general, the sequences of MADS-box proteins can be divided into four characteristic domains: the common MADS (M) domain, intervening (I), keratin-like (K), and C-terminal (C) domains (Cho et al., 1999). MIKC genes contain an intervening (I) domain of approximately 30 amino acids, which helps standardize dimerization reactions with poor conservation. The keratin-like (K) domain is a conserved region with a helical structure that facilitates interactions with proteins. The C-terminal (C) domain, being the least conserved, may function as a trans activation domain to stabilize protein–protein interactions (Davies et al., 1996; Riechmann et al., 1996; Kaufmann et al., 2005). The Type I MADS-box transcription factors in *Arabidopsis thaliana* can be subdivided into four groups, Mα, Mβ, Mγ and Mδ (Parenicova et al., 2003). Only a few type I genes possess biological functions, and knowledge of type I gene functions remains limited. Most evidence indicates that MADS-genes are related to many significant physiological and developmental processes of reproductive organs (Sheng et al., 2019). The famous ABC model, first proposed by Coen and Meyerowit, illustrates that the different steps of floral development are determined by three classes of homeotic genes (A, B and C), which confer identity to the four types of floral organs. However, many phenomena cannot be explained by the ABC model, and the complete ABCDE model was subsequently developed (Coen and Meyerowitz, 1991; Zahn et al., 2006; Silva et al., 2015). The ABCDE model is subdivided into five classes of genes (Parenicova et al., 2003; Irish, 2017). Class A genes specify sepal development, class B, C and E genes specify stamen development, and class D genes function alone to form ovules. In addition, the combination of class A, class B and class E genes is necessary for petals, while the combination of class C and class E genes determines the development of carpels. These five classes of genes are the main MADS-box genes. In *A. thaliana*, *APETALA1* (*AP1*) expressed in sepals and petals belongs to class A (Alejandra Mandel et al., 1992; Wang et al., 2014; Silva et al., 2015). The class B genes *PISTILLATA* (*PI*) and *APETALA3* (*AP3*) are expressed in petals and stamens (Jack et al., 1992). *AGAMOUS* (*AG*) is a representative class C gene expressed in stamens and carpels (Ditta et al., 2004; Ma, 2005). The class D gene *AGAMOUS-LIKE 11* (*AGL11*) is expressed in ovules. Class E genes, such as *SEPALLATA 1* (*SEP1*), *SEPALLATA 2* (*SEP2*), *SEPALLATA 3* (*SEP3*) and *SEPALLATA 4* (*SEP4*), are expressed in all floral whorls. Four other MADS-box transcription factors (*AGAMOUS-LIKE 24* (*AGL24*), *SUPPRESSOR OF OVEREXPRESSION OF CO 1* (*SOC1*), *FLOWERING LOCUS C* (*FLC*) and *SHORT VEGETATIVE PHASE* (*SVP*)) regulate flowering time. Among them, *AGL24* and *SCO1* promote flowering, while *FLC* and *SVP* inhibit flowering (Michaels et al., 2003). In addition, the MADS-box gene *RIPENING INHIBITOR* (*RIN*) regulates tomato fruit ripening (Wang et al., 2019).

*Juglans mandshurica* is a deciduous tree species grown in northern and northeastern China, Japan, Korea and the far eastern section of Russia. It is a precious fruit material and timber tree with significant economic, nutritional and medicinal value (Yan et al., 2021). Flower formation is an important process of the higher plant life cycle, and flowering at the right time ensures normal pollination and seed development, thereby controlling plant yield and quality. Therefore, the study of flower formation plays a decisive role in the development and utilization of *J. mandshurica*. Previous studies have shown that MADS-box family genes in *Solanum lycopersicum* (Wang et al., 2019), *Oryza sativa* (Arora et al., 2007), *Brassica rapa* (Duan et al., 2015), *Triticum aestivum* (Tardif et al., 2007), *Musa acuminata* (Liu et al., 2017), and *Malus domestica* (Velasco et al., 2010) are mainly involved in the regulation of floral organ and fruit development. However, the comprehensive analysis of MADS-box genes in *J. mandshurica* remains unclear. In our study, the genome-wide MADS-box genes in *J. mandshurica* were identified, the physical and chemical characteristics, chromosomal locations, phylogenetic relationship, gene structures, conserved motifs, and syntenic relationships were analyzed, and the expression patterns of key genes in female and male flowers were detected. Our results enhance the knowledge of the MADS-box gene family, which will lay a foundation for understanding the development of female and male flowers in *J. mandshurica*.

## Materials and methods

### Plant materials

The female and male flowers of *J. mandshurica* during the dormant bud, bud formation and flowering stages were obtained from Northeast Forestry University (Harbin, China N: 45°43′6.53″, E: 126°37′57.28″) in May 2021 and were named FS1, FS2 and FS3 and MS1, MS2 and MS3, respectively. The average high temperature is 12°C and low temperature is 1°C. The annual precipitation is 586.98 mm, and annual evaporation is 659.8 mm. These samples were frozen in liquid nitrogen and then stored at -80°C for RNA extraction.

### Identification of *JmMADS* genes

To perform genome-wide identification of the *JmMADS* gene family, the whole genome sequences and annotations were accessed from the NCBI database (https://www.ncbi.nlm.nih.gov/). The sequences of *Arabidopsis* MADS-box genes were obtained from the TAIR database (https://www.arabidopsis.org/browse/genefamily/MADSlike.jsp). A total of 107 *AtMADS* genes were used as reference sequences to identify *JmMADS* genes using the basic local alignment search tool-protein (BlastP). Each MADS-box subfamily had at least one representative MADS-box domain. Redundant sequences with the same chromosome locus were removed to ensure that the candidate genes were mapped to unique loci in their respective genomes. All identified *JmMADS* proteins were analyzed by the ExPASy program for molecular weight and isoelectric points. (https://web.expasy.org/compute_pi/). The subcellular localization was predicted using the Cell-PLoc 2.0 web tool (http://www.friendbio.com/meansMore/id/52).

### Phylogenetic analysis of *JmMADS* proteins

The MADS-box proteins of *A. thaliana* and *J. mandshurica* were subjected to multiple sequence alignment by the ClustaIW program of BioEdit. The phylogenetic tree was constructed using MEGA ver. 7.0 (neighborjoining algorithm, 1000 bootstrap replications).

### Analyses of gene structure and conserved motifs

The *J. mandshurica* MADS-box coding domain sequences (CDSs) and genomic DNA sequences were obtained from the National Center for Biotechnology Information (NCBI, http://www.ncbi.nlm.nih.gov/) database to analyze gene structure. MEME was used to analyze motifs. (Hu et al., 2010). The parameter was set to 10 conservative motifs and any number of repetitions. The final file was generated by TBtools.

### Analyses of chromosome location, collinearity, and gene duplication

The physical genome annotation files of *J. mandshurica* MADS-box genes were obtained from the NCBI database. TBtools was used to locate and visually map *JmMADS* genes on 16 chromosomes. To further analyze gene replication, duplicated and orthologous pairs of *JmMADS* genes were obtained by constructing a dual synteny plot in MCScanX (The Multiple Collinearity Scan toolkit X (MCScanX, http://chibba.pgml.uga.edu/mcscan2/), and Advanced Circos software (https://github.com/CJChen/TBtools) was used to display the collinearity circle plot. Homology between *J. mandshurica* and five representative species, including *J. rega*, *J. sigillata*, *P. trichocarpa*, *A. thaliana* and *V. vinifera*, was evaluated using MCScanX with the default parameters.

### Analysis of cis-acting elements in *JmMADS* promoters

Based on the *J. mandshurica* genome, the 2000 bp sequences upstream of the MADS-box gene CDSs were obtained as promoters to explore cis-acting elements using Plantcare (http://bioinformatics.psb.ugent.be/webtools/plantcare/Html/).

### Gene expression analysis of *JmMADS* genes

The RNA-seq data of *J. mandshurica* female and male flowers were obtained in the SRA database from the National Center for Biotechnology Information (NCBI; http://www.ncbi.nlm.nih.gov/) under the accession number PRJNA805360. After quality control, alignment and quantitative analysis, the expression levels of *JmMADS* genes were represented using reads per kilobase per million (RPKM). The data were normalized to obtain the relative expression values of all *JmMADS* genes involved in the development of floral organs in *J. mandshurica*, which were used to generate a heatmap by the heatmap program in TBtools.

### Total RNA extraction and qRT−PCR analysis

Total RNA was extracted from female and male flowers from three different stages using the DP441 RNAprep Pure Plant Plus Kit (Tiangen Biotech, Beijing, China). After assessing the quantity and quality, the cDNA was synthesized using the PrimeScript RT reagent Kit with gDNA Eraser (TaKaRa, Kyoto, Japan). The primers were designed by Primer3 web tools (version 4.1.0; https://primer3.ut.ee/). *18S* was used as an internal reference gene (**Table S5**). qRT−PCR was performed using an ABI PRISM 7500 Real-Time PCR system (Applied Biosystems, Foster City, USA). The gene expression levels were calculated using the 2 ^-ΔΔCT^ method (Li et al., 2021).

## Results

### Identification and analysis of *JmMADS* genes

To extensively identify the MADS-box family genes in *J. mandshurica*, the sequence alignment of MADS-box genes in *J. mandshurica* and *A. thaliana* was performed. After removing redundant sequences, a set of 67 highly homologous *J. mandshurica* MADS-box genes with completely functional structures were identified. These *JmMADS* genes were named *JmMADS1* to *JmMADS67* according to the gene annotation information (**Table S1**). The molecular characteristics, including protein molecular weight, isoelectric point, coding sequence length, and subcellular localization, were analyzed. Among these MADS-box genes, the shortest *JmMADS9* encoded 61 amino acids, while the longest *JmMADS39* encoded 387 amino acids. The protein molecular weight ranged from 7082.24 kDA (*JmMADS9*) to 42666.53 kDA (*JmMADS33*), and the isoelectric point ranged from 5.19 (*JmMADS6*) to 11.64 (*JmMADS18*). In addition, all MADS proteins were projected to be located in the nucleus. These 67 identified *JmMADS* genes were used for further analysis.

### Phylogenetic analysis of *JmMADS* proteins

**Figure 1:**
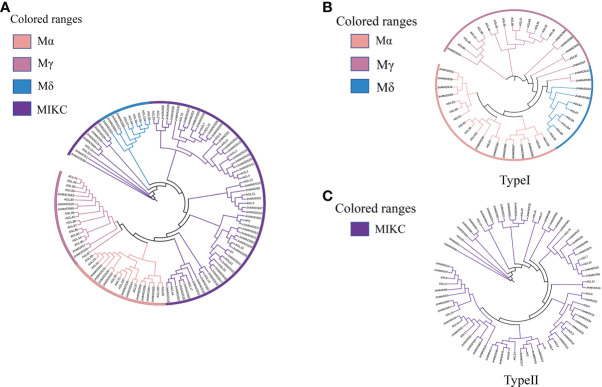
Phylogenetic trees. **(A)** Phylogenetic relationship of MADS-box proteins between *J. mandshurica* and *A. thaliana*. Four classes are represented by branches with different colors, including Mα (light pink), Mγ (deep pink), Mδ (blue), and MIKC (purple). Phylogenetic tree of type I **(B)** and type II **(C)** MADS-box domain proteins in *J. mandshurica* plants. The phylogenetic tree was constructed using MEGA 7.0 software and the neighbor-joining method with the following parameters: bootstrap analysis of 1,000 replicates, Poisson model, and pairwise deletion.

The phylogenetic analysis of *JmMADS* was performed based on the MADS-box proteins in *A. thaliana*. The 67 *JmMADS* proteins were classified into two types: type I (19 proteins) and type II (48 proteins) (**Figure 1A**). Based on the study of tomato, type I was subdivided into three subgroups (Mα, Mγ, and Mδ), of which Mα contained ten proteins. Mγ and Mδ contained four and five proteins, respectively (**Figures 1A, B**). Type II (MIKC) could be further divided into MIKC*- and MIKC^C^-type proteins. The MIKC^C^-type proteins contained the AP3/PI, SVP/AGL22, AGL15, AGL6, AP1/AGL7, FLC/AGL25, MADS-box proteins demonstrated the phylogenetic relationship and potential molecular function of the *JmMADS* gene family.

### Conserved motif and gene structure of *JmMADS*

**Figure 2:**
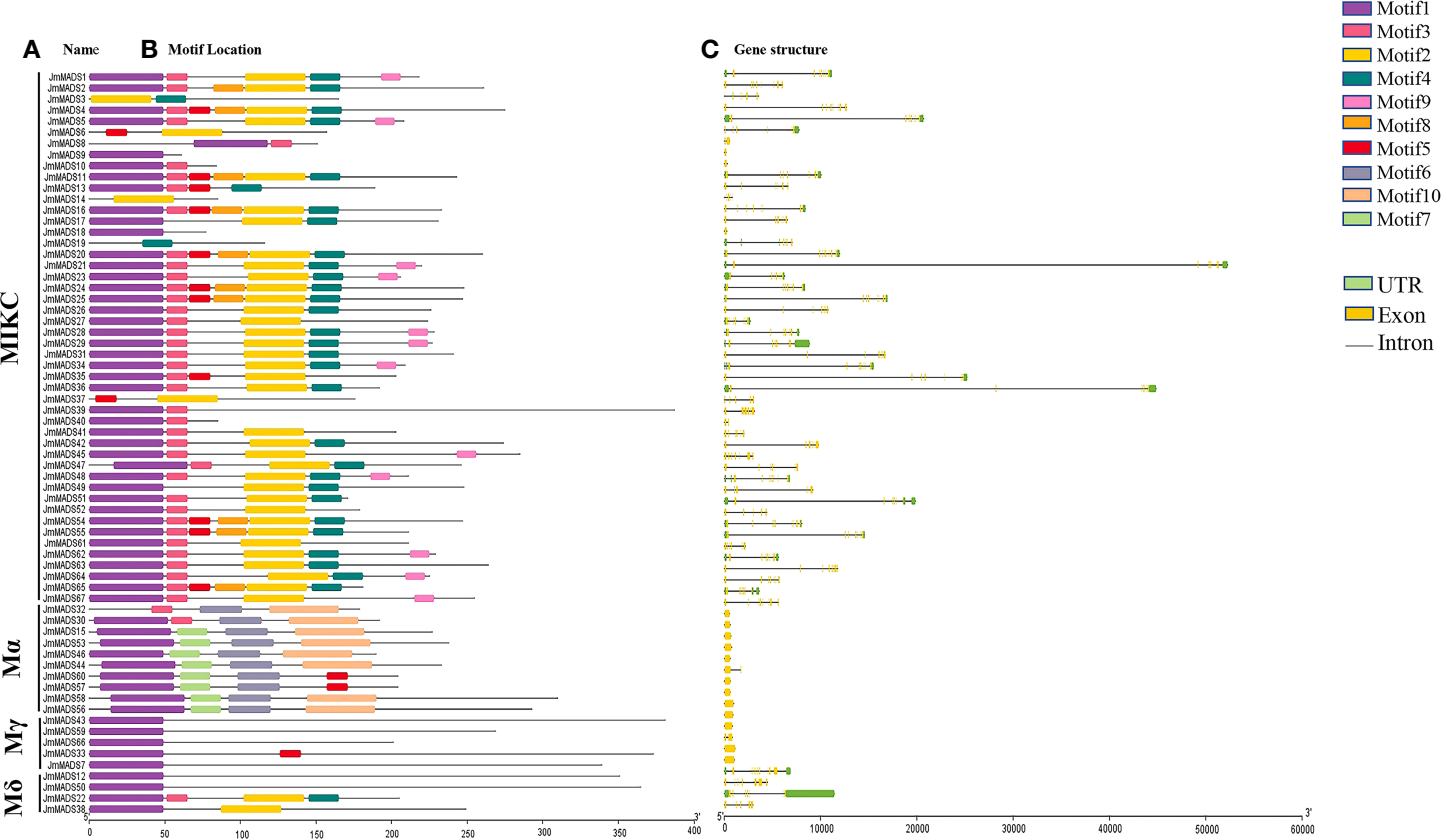
Analysis of gene structure and protein motifs. **(A)** The names of the *JmMADS* genes. **(B)** The motifs of the *JmMADS* proteins. Motifs 1–10 are displayed using different colored boxes listed. **(C)** The gene structure of *JmMADS* genes. Yellow boxes represent exons, black lines indicate introns, and green boxes represent the untranslated 5′ and 3′ regions.

Motifs are conserved sequences possessing biological functions, and different motifs usually have specific functional domains. A total of 10 conserved motifs, named motifs 1 to 10, were identified from the *JmMADS* proteins. Among these motifs, motif 1 was one of the most typical MADS-box domains found in *J. mandshurica* MADS-box proteins, excluding *JmMADS3*, *JmMADS6*, *JmMADS14*, *JmMADS19*, *JmMADS37*, and *JmMADS32*. Motifs 2, 3, and 4 were found in most MIKC genes, whereas motif 8, exhibiting poor conservation in *J. mandshurica*, was only found in MIKC MADS-box proteins. *JmMADS6* and *JmMADS37* only contained two motifs, that is motif 2 and motif 5. Furthermore, motifs 6, 7 and 10 were specific to the Mα proteins. In the Mγ and Mδ proteins, genes only have one motif except for *JmMADS33*, *JmMADS22* and *JmMADS38*. Besides, *JmMADS33* contained motif 1 and motif 5, *JmMADS38* contained motif 1 and motif 2 (**Figure 2A**). We speculated that the differences of conserved motif patterns might be related to the specific functions of proteins.

Structural differences in genes are the basis of gene family evolution, which contributes to the understanding of plant gene diversity and environmental adaptability. The analysis of *JmMADS* gene structure showed that the number of exons and introns in different *JmMADS* genes exhibited significant differences. The number of exons ranged from one to ten, while the number of introns ranged from zero to 11. *JmMADS12* contained the most exons (ten exons) and introns (11 introns). The Mα and Mγ groups of type I usually contained none or one intron. Some *JmMADS* genes, such as *JmMADS39*, *JmMADS40*, *JmMADS41*, *JmMADS42*, and *JmMADS45*, contained exons and introns but no UTR regions (**Figure 2C**). All these results showed that most genes belonging to the same type had similar genetic structures and conserved motifs.

### Chromosomal distribution and synteny analyses of *JmMADS* genes

**Figure 3:**
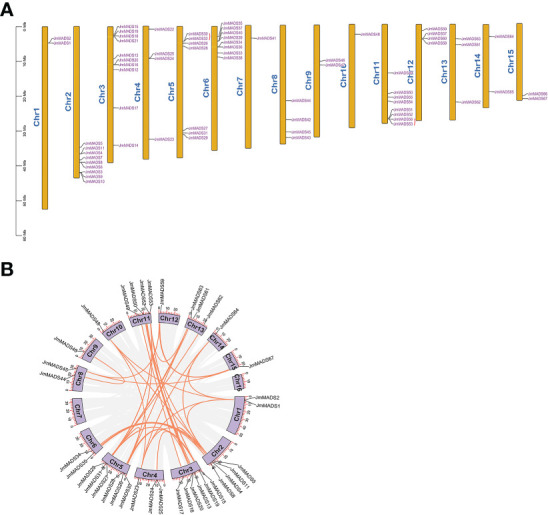
The chromosomal distribution and synteny blocks of *JmMADS* genes. **(A)** The 15 chromosomes (chromosomes 1 to 15) of *J. mandshurica* labeled with *JmMADS* gene names. Tandemly duplicated genes are marked with red arcs. **(B)** Genomic localization and gene duplication of *JmMADS* genes on *J. mandshurica* chromosomes. Gray lines indicate all syntenic blocks in the *J. mandshurica* genome; orange lines indicate the interchromosomal relationships of *JmMADS* genes.

The physical locations of *JmMADS* genes were mapped to the 15 chromosomes of *J. mandshurica*. The results showed that chromosome 3 contained the largest number of *JmMADS* genes (10 genes), followed by chromosome 2, which contained 9 genes. Chromosomes 7 and 10 only contained one *JmMADS* gene. A total of 2 (~3%), 4 (~6%), 7 (~10%), 8 (~12%), 4 (~6%), 2 (~3%), 8 (~12%), 4 (~6%), 3 (~4%), 2 (~3%), and 2 (~3%) genes were located on chromosomes 1, 4, 5, 6, 8, 9, 11, 12, 13, 14, and 15, respectively. (**Figure 3A**). Two tandem duplication gene pairs were obtained, which were located on chromosomes 5 and 11 (**Table S2**). These results showed that MADS-box genes were not evenly distributed on each chromosome, indicating the diversification and complexity of the MADS-box gene family.

To understand the duplication events of all *JmMADS* genes, we performed synteny analysis using MCscanX and Advanced Circos software in TBtools. A total of 48 pairs of duplicated genes were distributed in 15 of 16 *J. mandshurica* chromosomes. In addition, syntenic genes distributed on chromosome 3 and chromosome 5 (6 gene pairs) were the most common, followed by chromosome 2 and chromosome 11 (4 gene pairs). Nevertheless, only one duplication gene pair was found on chromosomes 9, 10 and 15. In addition, there were no duplicates on chromosomes 7 and 16 (**Figure 3B**). These results demonstrated that replication events were the fundamental driving force of MADS-box gene evolution.

### Syntenic relationships between *JmMADS* genes and MADS-box of other species

**Figure 4:**
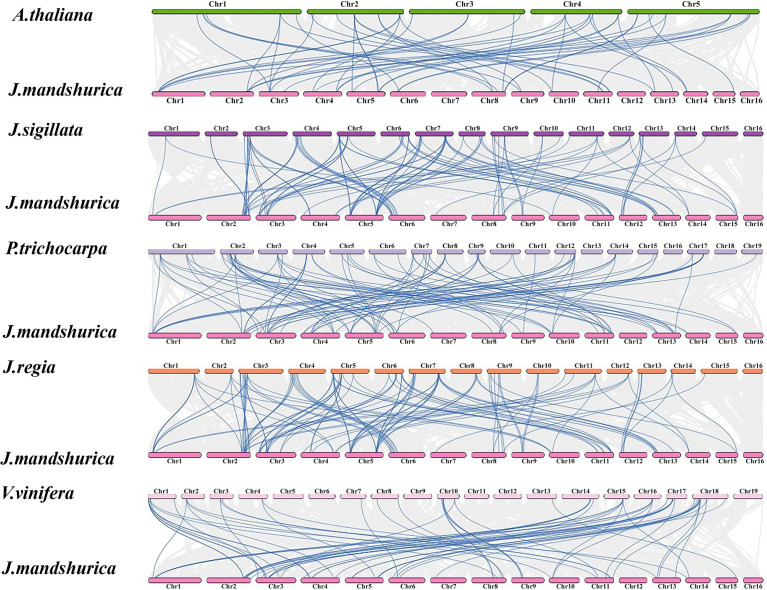
Synteny analysis of the MADS-box genes between *J. mandshurica* and *A. thaliana*, *J. sigillata*, *P. trichocarpa*, *J. regia*, and *V. vinifera*. The gray lines indicate gene blocks in poplar that are orthologous to other genomes. The blue lines delineate the syntenic MADS-box gene pairs.

The collinear relationships of MADS-box genes between *J. mandshurica* and several reported species, including *A. thaliana* (Cheng et al., 2017), *Juglans sigillata* (Ning et al., 2020), *Populus trichocarpa* (Tuskan et al., 2006), *Juglans regia* (Peng et al., 2017), and *Vitis vinifera* (Zhou et al., 2017), were explored based on evolutionary relationships. The syntenic gene pairs on chromosome 5 were the most abundant and diverse according to the intra- and interspecies collinearity analysis of 67 *JmMADS* genes. *J. mandshurica*, *J. sigillata*, and *J. regia* exhibited the highest level of homology and great collinearity, and the largest number of orthologous gene pairs in *J. mandshurica* and *J. sigillata* were distributed on chromosomes 5 and 7. *J. mandshurica* and *P. trichocarpa* also exhibited higher homology, and their orthologous gene pairs were mainly distributed on chromosomes 5 and 2, respectively. *J. mandshurica* and *A. thaliana* (orthologous gene pairs were mainly distributed on chromosome 2) exhibited relatively low homology (**Figure 4**). The results indicated that *J. mandshurica* MADS-box genes were closely related to other MADS-box members in the five mentioned plant species.

### Analysis of cis-acting elements in *JmMADS* promoters

**Figure 5:**
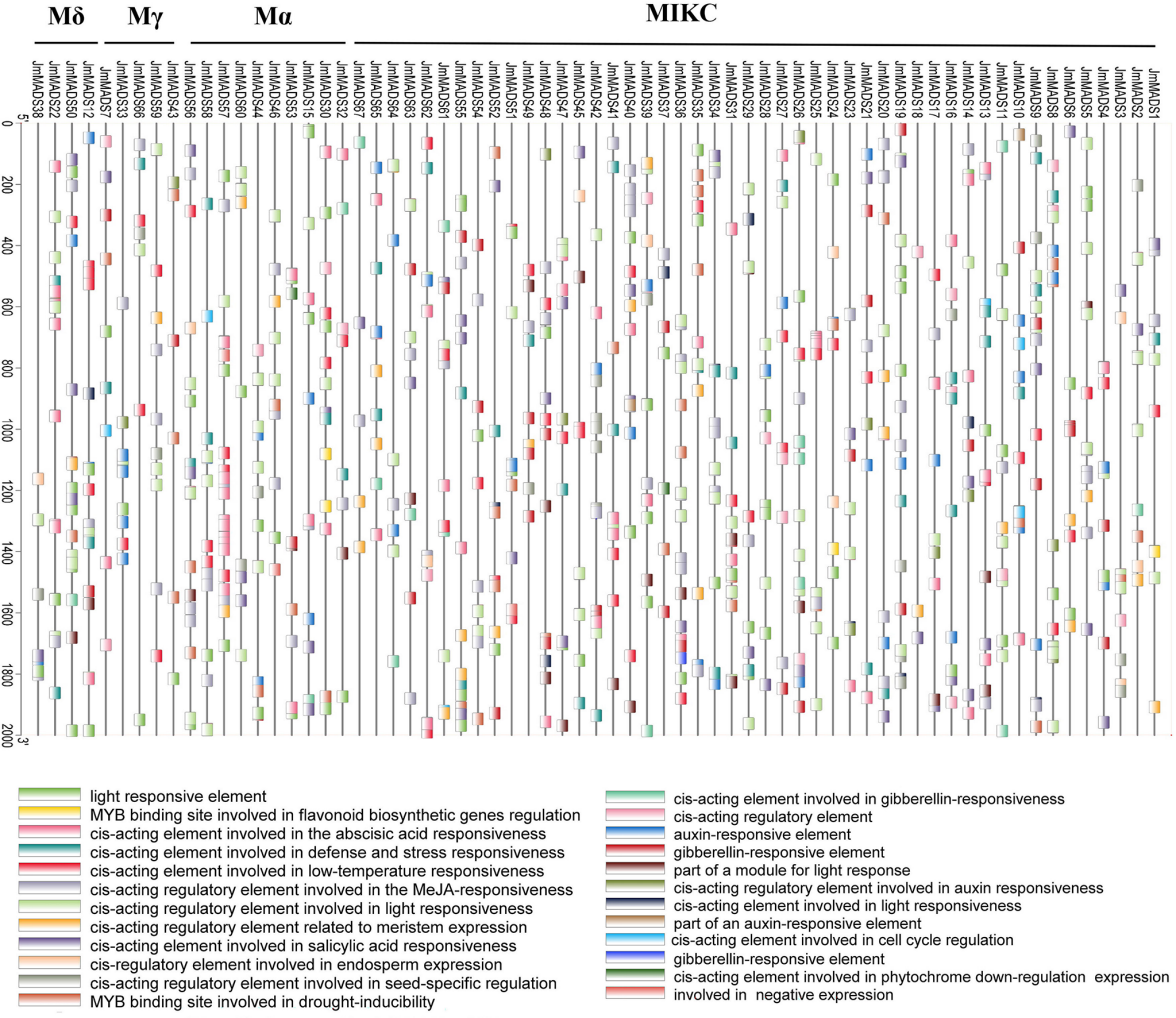
Analysis of cis-acting elements of MADS-box genes. *JmMADS1*~ *JmMADS67* represent 67 genes in *J. mandshurica*; Mδ, Mγ, Mα, MIKC represent the four subfamilies. Boxes with different colors at the bottom represent the cis-acting elements.

Cis-acting elements are binding sites of transcription factors that regulate gene expression responsible for the growth, differentiation, and development of plants. The cis-acting elements of 2000 bp sequences in the MADS-box gene promoter regions were explored. A total of 24 cis-acting elements were obtained according to functional annotations; eight of them were associated with hormone response, and five were associated with light response. Interestingly, only *JmMADS1*, *JmMADS24*, and *JmMADS30* contained cis-acting, MYB binding sites involved in flavonoid biosynthesis gene regulation (**Figure 5**). The analysis of cis-acting elements showed that most of the MADS-box genes might be associated with stress, light or hormone responses.

### Gene expression patterns of *JmMADS* genes during floral organ development

**Figure 6:**
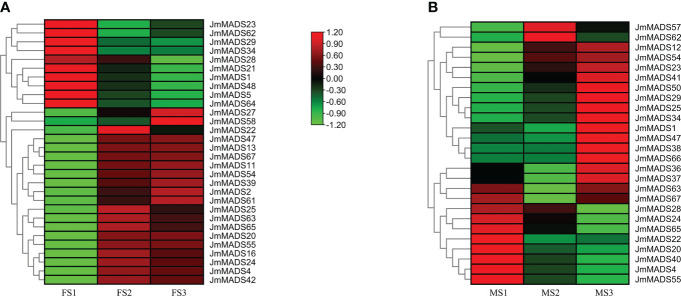
Expression patterns of *JmMADS* genes in different stages of floral organ development. **(A)** Heatmap of 30 *JmMADS* genes in female flowers at the dormant bud (FS1), bud formation (FS2), and flowering (FS3) stages. **(B)** Heatmap of 26 *JmMADS* genes in male flowers at the dormant bud (MS1), anther formation (MS2), and anther maturation (MS3) stages.

The MADS-box gene family plays an important role in floral organ development. The expression patterns of *JmMADS* genes at different stages (including female flower development from the FS1 to FS3 stages and male flower development from the MS1 to MS3 stages) were analyzed. Among the identified genes, a total of 30 and 26 *JmMADS* genes were specifically expressed in the female and male flowers, respectively. Seventeen *JmMADS* genes were commonly expressed in the two tissues, and transcriptome data of 28 genes were not available. In female flowers, 10, 19, and 19 *JmMADS* genes were significantly upregulated in the FS1, FS2, and FS3 stages, respectively (**Figure 6A**). In male flowers, 10, 6, and 16 *JmMADS* genes exhibited higher expression levels in the MS1, MS2, and MS3 stages (**Figure 6B**). Most type II *JmMADS* genes exhibited particularly high expression during flower development. These results indicated that the MADS-genes had different expression levels during different stages of flower organ development in *J. mandshurica*.

**Figure 7:**
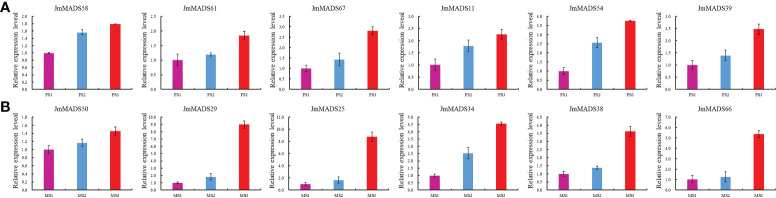
The expression levels of 12 *JmMADS* genes during the development of female and male flowers. **(A)** The expression of 6 *JmMADS* genes in female flowers using RT−qPCR. **(B)** The expression of 6 *JmMADS* genes in male flowers using RT−qPCR. The y-axis shows the gene expression levels detected by RT−qPCR. The x-axis represents the tissues of different stages. The error bars represent standard error. The values are the mean ± standard deviation of three replicates.

To further investigate the function of *JmMADS* genes in floral organ development, the expression of 12 genes selected from three developmental stages of female and male flowers was detected in *J. mandshurica* female and male flowers by qRT−PCR (**Figure 7**). All selected *JmMADS* genes were upregulated from the FS1 to FS3 stages in female flowers and from the MS1 to MS3 stages in male flowers. The expression levels of *JmMADS29*, *JmMADS25*, *JmMADS34*, *JmMADS38*, and *JmMADS66* in the MS3 stage were significantly higher than those in the MS1 and MS2 stages. The gene expression measured by qRT−PCR showed similar patterns to that detected by RNA-seq. These results indicated positive roles of *JmMADS* genes during the development of female and male flowers.

## Discussion

*J. mandshurica* is a widespread broad-leaved tree species whose seed kernel has high nutritional and medicinal values (Li et al., 2022). TFs possess diverse functions, such as controlling plant growth and development and responding to biotic and abiotic stresses. MADS-box transcription factors are one of the plant-specific TF families and contribute to floral organ specification, flowering time regulation, fruit development and ripening (Lai et al., 2022). Genome-wide identification and analysis is an essential method to study the specific functions of species gene familys (Rounsley et al., 1995; Becker and Theißen, 2003). Presently, a total of 107, 105, 32, 71, 43, 146, 131 and 48 MADS-box family members were identified from Arabidopsis (Parenicova et al., 2003; Smaczniak et al., 2012), poplar (Leseberg et al., 2006), rice (Arora et al., 2007), grape (Díaz-Riquelme et al., 2009), cucumber (Hu and Liu, 2012), apple (Tian et al., 2015), tomato (Wang et al., 2019), and pineapple (Zhang et al., 2020). However, the MADS-box gene family of *J. mandshurica* has not been systematically analyzed. Our study identified 67 MADS-box proteins of *J. mandshurica* (**Table S1**). These studies suggested that the gene retention duplication differed across species, resulting in different numbers of MADS-box genes in distinct species with different evolutionary constraints. The genome-wide identification and analysis of the MADS-box gene family in *J. mandshurica* provided important genetic information for lightning the development of *J. mandshurica* female and male flowers.

The number and structural characteristics of TFs are determined by the genome size and the long-term evolutionary impact of plants. In the present study, the phylogenetic relationships between 67 *J. mandshurica* and *A. thaliana* MADS-box proteins were analyzed. The results showed that the *J. mandshurica* MADS-box proteins were divided into two types (type I and type II) containing four subgroups (MIKC, Mα, Mγ, and Mδ) (**Figure 1**). Compared with *A. thaliana*, a relatively lower number of MADS-box proteins were found in *J. mandshurica*. A total of 19 *JmMADS* genes were classified as type I genes, divided into the Mα, Mγ, and Mδ subgroups, while 48 *JmMADs* genes were identified as type II genes (MIKC^C^). The results suggested that *J. mandshurica* type II MADS-box genes might have a lower gene loss rate after duplication and/or a higher duplication rate. These two types might evolve differently. The type I MADS-box genes might be mainly caused by small-scale duplication, whereas type II genes were caused by whole-genome duplication. Type II contained more *JmMADS* and *AtMADS* proteins than type I (**Figures 1B, C**), which might also be caused by the rapid existence and extinction of type I MADS-box genes. In addition, the previous study indicated that MIKC^C^ genes, even including complete subgroups of MIKC^C^ genes, had been found in many plant species, and the number of MIKC* genes in some plants was also lower than that of MIKC^C^ genes, The results indicated that the MIKC^C^ subgroup genes were conserved in different species, while other subgroups were not (Tian et al., 2015).

All 67 *JmMADS* proteins were predicted to be located in the nucleus, which might be closely related to the regulation of gene expression mediated by TFs (**Table S1**). The analysis of *J. mandshurica* MADS-box gene structural features showed that the exons of all identified *JmMADS* genes ranged from one to ten, and the introns ranged from zero to 11 (**Figure 2C**), demonstrating the complex functional differentiation and structural diversity of *JmMADS* genes. The Mα and Mγ subgroups of the type I genes usually had no or one intron, exhibiting the potential of specific evolutionary processes, which might be promoted by the loss of multiple introns during MADS-box gene family diversification. Similar results were found in tomatoes (Wang et al., 2019).

In addition, the intron numbers of type II and Mδ (type I) *JmMADS* genes were greater than those of Mα and Mγ subgroup genes, indicating the high conservation of type II and Mδ (type I) *JmMADS* genes. The results were consistent with the MADS-box gene structure analysis in tomato (Wang et al., 2019). Ten conserved motifs were found in *JmMADS* proteins, and all *JmMADS* proteins contained motif 1, excluding *JmMADS*3, *JmMADS*6, *JmMADS*14, *JmMADS*19, *JmMADS*37, and *JmMADS*32 (**Figure 2A**). The different motifs usually have specific functional domains, and our results suggested that the conserved motif might play an important role in the specific functions of the MADS-box gene family. All analyses of the *JmMADS* gene structure and conserved motifs could provide more valuable information for the evolutionary relationship of this gene family.

Gene duplication universally forces genome complexity in eukaryotic families (Hughes, 1994), which is also an evolutionary event of many plant species. Tandem replication is considered the duplication of more than two genes on one chromosome, which is beneficial for the rapid expansion and formation of gene families. Previous studies have shown that gene duplication events have occurred in many gene families, such as MYB, NAC, and WRKY TFs, to avoid extinction. In this study, *JmMADS* genes were randomly distributed over 15 of 16 *J. mandshurica* chromosomes, and one tandem duplication gene pair was separately identified on chromosomes 5 and 11 (**Figure 3A**). This phenomenon might help *J. mandshurica* evolve different characteristics from other plants, thereby contributing to diverse gene functions. In addition, a total of 48 orthologous gene pairs were found among 67 identified *JmMADS* genes using synteny analysis, indicating that the species evolution of *J. mandshurica* was associated with gene relationships. The *JmMADS*1, *JmMADS*5, *JmMADS*23 and *JmMADS*26 genes had multiple orthologous gene pairs (**Figure 3B**), which have been further confirmed their crucial roles in plant growth and adaptation (Li et al., 2021; Lai et al., 2022).

Plant promoters contain important regulatory elements that affect gene transcription (Huang et al., 2021; Liu et al., 2022). The study of plant gene promoter functions can reflect the corresponding gene responses. Our study found similarities and differences in the different *JmMADS* promoters. Many cis-acting elements involved in the light response, stress response, low temperature response, hormone response, gibberellin response and auxin response were identified from the promoter regions of *JmMADS* genes (**Figure 5**). The existence of these cis-acting elements indicated that the MADS-box genes responded to abiotic stress and hormonal stimulation. These results further support the study of *JmMADS* gene functions.

The flower organ development is a very important stage of the plant life cycle. With the rapid development of molecular biology, studies on the regulatory mechanism of plant flower development at the molecular level have received increasing attention. Previous studies have found that many transcription factors play a fundamental role in the identification of flower organs (Ma, 1994). Specifically, MADS-box transcription factors. For example, the *PMADS4* and *PMADS12* genes have been identified to play an important role in the development of flowers in petunia (Immink et al., 2003). The MADS-box genes *TAG1* and *TAGL1* from the monophyletic *AGAMOUS* (*AG*) subfamily of tomato are mainly expressed in stamens and carpel (Gimenez et al., 2016). In addition, the protein complexes formed by SlMBP21 with JOINTLESS and MACROCALYX are transcriptional activators of tomato flower abscission zone development. Furthermore, a majority of pineapple MADS-box genes are highly expressed in flowers, which suggests that MADS-box genes of pineapple are closely related to flowering development (Zhang et al., 2020). However, the regulatory function of MADS-box transcription factors (TFs) in floral organ development in *J. mandshurica* is still unclear. In our study, all MADS-box genes of *J. mandshurica* exhibited different expression patterns in different growth and development stages (**Figure 6**). Among the 67 MADS-box genes, expression analyses identified a total of 39 *J. mandshurica* MADS-box genes that displayed diverse patterns of transcript accumulation in female and male flowers in different floral development stages. Furthermore, 19 key *JmMADS* genes were significantly up-regulated in FS3 stages in *J. mandshurica* female flowers, and 16 key *JmMADS* genes were up-regulated in male flowers in MS3 stages, suggesting that they had specific regulation on the development of female and male flowers. Furthermore, *JmMADS*58 and *JmMADS*61 were significantly upregulated in the FS3 stage, and *JmMADS*50 and *JmMADS*29 were also significantly upregulated in MS3. These genes might be positive regulators to support metabolic activities concerning floral organ development with redundant functions. In addition, we also identified 12 critical *JmMADS* genes that exhibited constitutively high expression levels during ripening stages in *J. mandshurica* female and male flowers, and the results were verified by RT−qPCR analysis (**Figure 7**). Therefore, we inferred strict regulation of flower development. The MADS-box genes of *J. mandshurica* validation in this study will help us understand the role of MADS-box genes in the development of female and male flowers and will lay a foundation for us to further explore the genetic function of MADS-box genes in floral organs.

## Conclusion

In conclusion, a total of 67 MADS-box genes in *J. mandshurica* were identified and divided into type I (19 genes) and type II (48 genes). Type I was subdivided into three subgroups (Mα, Mγ, and Mδ), and type II was MIKC^C^-type based on phylogenetic relationship. Most genes belonging to the same type had similar gene structures and conserved motifs. The MADS-box genes were not evenly distributed on each chromosome, and chromosome 3 contained the largest number of *JmMADS* genes. The syntenic gene pairs on chromosome 5 were the most abundant and diverse. The MADS-box genes in *J. mandshurica*, *J. sigillata*, and *J. regia* exhibited the highest homology and great collinearity. The promoters of MADS-box genes contained cis-acting elements related to hormone, light, and stress responses. In addition, 30 and 26 *JmMADS* genes were specifically expressed in the female and male flowers, of which 12 selected genes were significantly upregulated during floral organ development. Our results provide new insights into the regulatory functions of MADS-box genes during floral development in *J. mandshurica*, enhancing the understanding of the underlying evolutionary relationship and characteristics of the MADS-box gene family in tree species.

## Data availability statement

The datasets presented in this study can be found in online repositories. The names of the repository/repositories and accession number(s) can be found in the article/**Supplementary Material**.

## Author contributions

RH, GQ and XYZ designed and managed the research work and improved the manuscript; HL, YuL and QW performed the bioinformatics analysis; XXZ and KC performed the experiments; HL wrote the manuscript; HL and YaL prepared all figures. All the authors reviewed the manuscript. All authors contributed to the article and approved the submitted version.

## Funding

The work was supported by the Forestry Science Technology and Development Project (KJZXSA202208).

## Conflict of interest

The authors declare that the research was conducted in the absence of any commercial or financial relationships that could be construed as a potential conflict of interest.

## Publisher’s note

All claims expressed in this article are solely those of the authors and do not necessarily represent those of their affiliated organizations, or those of the publisher, the editors and the reviewers. Any product that may be evaluated in this article, or claim that may be made by its manufacturer, is not guaranteed or endorsed by the publisher.
